# Prevalence of mixed genotype hepatitis C virus infections in the UK as determined by genotype‐specific PCR and deep sequencing

**DOI:** 10.1111/jvh.12849

**Published:** 2018-02-21

**Authors:** A. L. McNaughton, V. B. Sreenu, G. Wilkie, R. Gunson, K. Templeton, E. C. M. Leitch

**Affiliations:** ^1^ MRC‐University of Glasgow Centre for Virus Research Glasgow UK; ^2^ West of Scotland Specialist Virology Centre Royal Infirmary of Glasgow Glasgow UK; ^3^ Edinburgh Specialist Virology Centre Edinburgh UK; ^4^ Present address: Nuffield Department of Medicine University of Oxford Oxford UK

**Keywords:** direct‐acting antivirals, HCV genotyping, hepatitis C virus, mixed genotype HCV infections, next‐generation sequencing

## Abstract

The incidence of mixed genotype hepatitis C virus (HCV) infections in the UK is largely unknown. As the efficacy of direct‐acting antivirals is variable across different genotypes, treatment regimens are tailored to the infecting genotype, which may pose issues for the treatment of underlying genotypes within undiagnosed mixed genotype HCV infections. There is therefore a need to accurately diagnose mixed genotype infections prior to treatment. PCR‐based diagnostic tools were developed to screen for the occurrence of mixed genotype infections caused by the most common UK genotypes, 1a and 3, in a cohort of 506 individuals diagnosed with either of these genotypes. The overall prevalence rate of mixed infection was 3.8%; however, this rate was unevenly distributed, with 6.7% of individuals diagnosed with genotype 3 harbouring genotype 1a strains and only 0.8% of samples from genotype 1a patients harbouring genotype 3 (*P* < .05). Mixed infection samples consisted of a major and a minor genotype, with the latter constituting less than 21% of the total viral load and, in 67% of cases, less than 1% of the viral load. Analysis of a subset of the cohort by Illumina PCR next‐generation sequencing resulted in a much greater incidence rate than obtained by PCR. This may have occurred due to the nonquantitative nature of the technique and despite the designation of false‐positive thresholds based on negative controls.

**Abbreviations**
DAA: direct acting antiviral
HCV: hepatitis C virus
NGS: next‐generation sequencing
PWID: people who inject drugs
SVR: sustained virological response

## 1 INTRODUCTION

The prevalence of mixed genotype HCV infections has been assessed across numerous geographical regions with broad estimates between 1.2% and 25.3% being reported.^1^, ^2^ Although some variability can be explained by study design differences, populations and detection methods, the numbers of mixed infection positive patients identified in studies are frequently too low to obtain meaningful statistical power.^3^ The incidence of mixed genotype HCV infections in larger studies is less than 8%.^4^, ^5^, ^6^, ^7^ In the UK, only a relatively small study has been conducted which indicated prevalence rates of 9% and 19% in PWID and haemophiliacs, respectively.^8^ Many tests currently used in clinical settings lack the sensitivity and specificity required for diagnosis of mixed infection which are therefore rarely detected.^5^

DAA therapies for HCV infection have revolutionised treatment of the disease. These new drugs have been optimised for genotype 1 (gt1), and SVR rates can be lower for other genotypes, particularly gt3. Consequently genotype‐specific regimens may be required for effective treatment.^9^ The efficacy of DAAs against mixed genotype infections has yet to be determined although several studies have hypothesized that mixed infections may only be partially resolved by therapy regimens tailored to the major infecting genotype, leading to genotype switching.^10^, ^11^

Due to morbidity issues and cost implications of repeated DAA treatments, improved diagnostics for mixed genotype infections to optimise treatment regimens are important. In this study, we sought to determine the prevalence of mixed HCV infection in a cohort of 506 HCV‐positive patients from across Scotland. We focused on gt1a and gt3 which together constitute greater than 90% of the total HCV infections in the UK. Highly sensitive genotype‐specific nested PCR assays were developed and used to screen for gt1a/gt3 mixed infections. The relative proportion of each genotype within the mixed infections was determined by real‐time (rt)‐PCR. Furthermore, we compared the genotype‐specific PCR techniques with NGS for diagnosis of mixed HCV infections.

## 2 MATERIALS AND METHODS

### 2.1 Patient cohort

Anonymised sera samples from 506 HCV‐positive patients genotyped as 1a or 3 were obtained from the West of Scotland and Lothian Specialist Virology Centres. Samples were acquired during 2011, 2013 and 2014. Ethical approval was granted by the NHS Research Scotland Greater Glasgow and Clyde Biorepository.

### 2.2 Control transcripts

Control RNA transcripts derived from synthetic dsDNA based on a gt1a (H77, AF009606) and a gt3 (3a.GB.2005, GQ356206) sequence were used for PCR and rt‐PCR optimisation, for relative quantification and as NGS fidelity controls.^3^, ^12^, ^13^, ^14^

### 2.3 RNA extraction and PCR

Viral RNA extracted from serum samples using a QIAamp viral RNA mini kit (Qiagen, Germantown, MD, USA) was transcribed into cDNA using SuperScript III reverse transcriptase (Thermo Fisher Scientific, Waltham, MA, USA). Nested PCR was performed using Platinum Taq (Thermo Fisher Scientific) and KOD Hot Start (Merk Millipore, Billerica, MA, USA) sequentially for 40 and 25 cycles, respectively. Primers used to screen for gt1a and gt3 strains (Table 1) were designed using alignments of reference sequences from the Los Alamos HCV database.^15^ Screening reactions included a transcript of the appropriate genotype (positive control) and a negative control consisting of water replacing sample. Amplicons were treated with Illustra Exostar (VWR Int., Radnor, PA, USA) and Sanger‐sequenced. Mock mixed infections consisting of gt1a and gt3a transcript control RNA were assessed. These controls contained 25, 50 or 100 copies/μL of the minor strain with 10^6^ copies/μL of the major genotype.

**Table 1:** Primers and probes designed for this study

Genotype specificity	Primer type	Sequence	Genome position^a^
PCR			
Gt1a	OS	CAT ATA ACG GGY CAY CGC ATG G	1275‐1296
	OAS	TGG TTY GGY TGY ACY TGG ATG AA	2008‐1986
	IS	ATG ATR ATG AAC TGG TCY CCY AC	1305‐1327
	IAS	TYG TCC TYA AYA ACA CYA GRC C	1972‐1951
Gt3	OS	TTY CTY GTG GGR CAA GCC TTC A	1203‐1224
	OAS	CCT YTW CTG CCC CAC YGA CTG	2143‐2123
	IS	TTY AGA CCY CGY CGC CAT CA	1227‐1246
	IAS	CAG AYG TGT TCY TGC TGR AGT C	1993‐1972
Pan‐genotypic	S	GC NTG GGA YAT GAT GAT GAA YTG	1296‐1318
	AS	GDG SGT ART GCC ARC ART ANG	1812‐1792
Real‐time PCR			
Gt1a	S	CTG TCG AGC CGC AGG GCT C	8507‐8525
	AS	GCT CCA AGT CGT AYT CTG GYT GBG	8686‐8662
	Probe	FAMTM‐CCT CCG TGA AGG CTC TCA GGY TCG CYG CG‐MGB	8625‐8597
Gt3	S	GGA ACC CGG ACT TYC TYG TCT G	8527‐8548
	AS	CTC AAG GTC RTA GGT RGG CTG YGG	8684‐8661
	Probe	FAMTM‐CGA CGC CRT CAC TCT CRG CCA CCA CRA CYA G‐MGB	8589‐8559

O, outer; I, inner; S, sense; AS, antisense.

a Genome position is relative to the HCV strain H77 (Kuiken et al., 2006).

### 2.4 Sequence analysis

Sequences were aligned using MUSCLE (v3.8) within SSE.^16^, ^17^ Maximum likelihood phylogenetic trees were produced using MEGA 5.0.^18^ Bootstrap resampling (1000 replicates) was used to assess statistical support for tree branches, with bootstrap values ≥70% considered significant. Reference sequences were downloaded from NCBI Genbank and the Los Alamos HCV Database.^19^, ^20^

### 2.5 rt‐PCR

rt‐PCR was performed with the TaqMan fast 7500 system using TaqMan fast reagents (Thermo Fisher Scientific). An rt‐PCR targeting the 5′ UTR was used to quantify the HCV viral load^21^ using a dilution series prepared from known concentrations of JFH‐1 replicon transcripts. Genotype‐specific rt‐PCR was performed using newly designed primers and probes targeting the NS5B region (Table 1).

### 2.6 Deep sequencing

In addition to the 19 mixed infection samples, 19 gt1a and 20 gt3 randomly selected samples determined by PCR screening to be mono‐infected also underwent deep sequencing. Mock mixed infections were prepared from gt1a and gt3a transcript control RNA consisting of 10^3^, 10^4^ or 10^5^ copies/μL of the minor strain with the major genotype to give a total of 10^6^ copies/μL. Aliquots of single genotype transcripts (10^6^ copies/μL) were used as fidelity controls, whereas HCV‐negative serum and H_2_O provided negative controls. To limit contamination risk, samples were sequenced in two runs on the basis of genotype or major genotype.

Pan‐genotypic PCR primers designed targeting partial E1‐E2 region (Table 1) were modified by a phosphorothioate bond between the final two 3′ end nucleotides to render them resistant to 3′ to 5′ endonuclease activity by proofreading enzymes. cDNA from screening reactions was amplified with the KAPA HiFi PCR kit (Sigma‐Aldrich, Dorset, UK) for 30 cycles. Amplicons purified by Agencourt AMPure XP magnetic beads (Beckman Coulter, Pasadena, CA, USA) were quantified using the Qubit dsDNA High‐Sensitivity kit (Thermo Fisher Scientific). Libraries prepared using the KAPA library preparation kit (Sigma‐Aldrich) were analysed by TapeStation (Agilent Technologies, Santa Clara, CA, USA) to assess product size. Samples were pooled in equimolar ratios, and denatured libraries were run using the V3 MiSeq reagent kit (Illumina, San Diego, CA, USA) for 300 bp paired‐end (p.e.) sequencing on a MiSeq Desktop Sequencer (Illumina). As diversity among samples was low, they were run at a relatively low cluster density with 5%‐10% PhiX controls.

### 2.7 Deep sequencing analysis

Deep sequencing data were analysed using an in‐house Unix‐based pipeline. Low‐quality reads were identified by FastQC and sequencing adapters removed using Trim Galore. An in‐house bioinformatics programme was developed for genotyping HCV using high‐throughput sequences. This comparative genotype assignment method took 37 bp k‐mers from sequence reads and compared them against a list of reference genotype‐specific k‐mers of the same length. Although a longer k‐mer could improve genotyping, they increase the risk of mismatches and require more computing power. Based on the breadth and depth of k‐mer coverage, genotypes were assigned to samples. The best references to map sequence reads were selected based on the genotyping programme, and reads were mapped to reference genomes using Tanoti.^22^ Consensus sequences were generated and compared with Sanger‐sequenced reads and reference genomes by phylogenetic analysis.

### 2.8 Statistical analyses

Differences in the means and distributions of data were compared using the independent samples t test function. The significance of differences in the distribution of categorical data was determined using Chi‐squared tests.

## 3 RESULTS

### 3.1 Patient characteristics

Mixed gt1a/gt3 HCV infections were detected in samples collected from individuals residing throughout Scotland, indicating that no geographical region was particularly associated with mixed infections. The average age was comparable (*P* = .84) between individuals with mixed infections (40.6 ± 9.7 years) and mono‐infected individuals (40.3 ± 9.3 years). The average viral load of mixed genotype infection samples was 5.78 ± 1.0 log_10_ IU/mL, similar (*P* = .61) to the average viral load observed in the cohort (5.63 ± 0.95 log_10_ IU/mL). Detailed clinical information was only available for patients residing within the Glasgow and Paisley postcode districts, accounting for 46.2% of the cohort. Within this group, 76.4% were male and a history of injecting drug use was recorded for 70.7% of subjects. Detailed clinical data were available for 4 individuals with mixed genotype infections. Liver disease was recorded for three of these subjects, two with cirrhosis and one with fibrosis. All four patients had a history of psychiatric disorders. Three of the patients had received treatment for HCV infection (treatment type unknown) and of these, one had not yet completed treatment and two had failed to achieve an SVR.

### 3.2 PCR analysis

PCR assay sensitivity was evaluated using known dilutions of the transcript controls tested in batches of 8 replicates with a negative control. Results were converted into probit values^14^, ^23^ and using these values, a 90% detection rate of 9 (gt1a) and 21 (gt3) copies/μL of RNA were calculated. The specificity of the PCRs was confirmed by amplification and sequencing of the minor transcripts within mock mixed infections.

**Figure 1:**
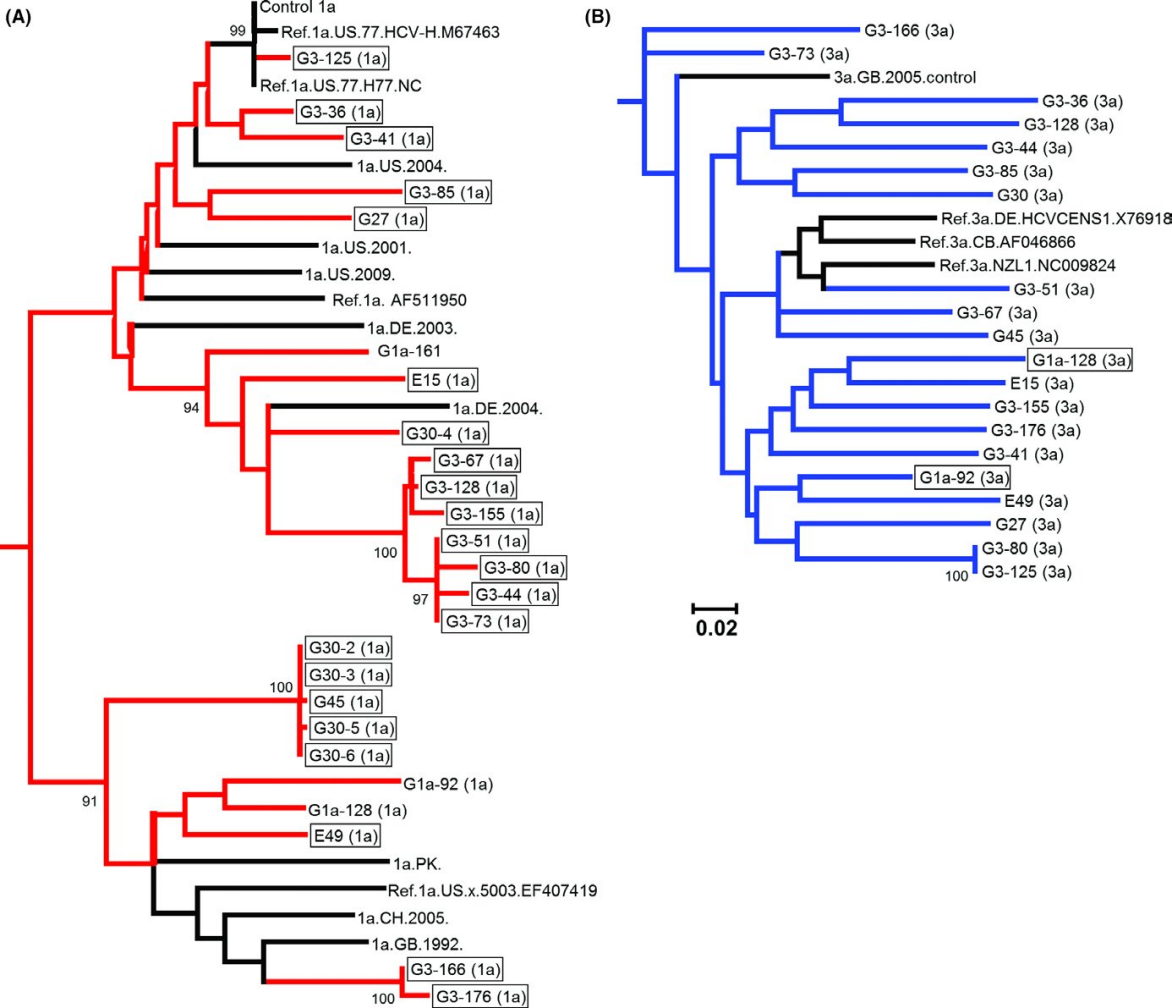
Maximum likelihood phylogenetic trees of A, gt1a sequences (red) and B, gt3 sequences (blue) obtained from samples with mixed genotype HCV infections. Reference sequences are shown in black. Sequences obtained from individuals diagnosed with the opposite genotype are shown in boxes. Bootstrap support of >70% after 1000 replicates is shown

A total of 506 patients diagnosed with gt1a or gt3 infection were screened for the presence of mixed gt1a/gt3 infection. Overall, 3.8% (19 of 506) of samples had a mixed gt1a/gt3 infection. Although 6.7% (17 of 252) of samples from gt3‐diagnosed patients also contained gt1a, in the corresponding screen of gt1a samples, significantly fewer patients (*P* < .05) had an undiagnosed secondary gt3 infecting strain (0.8%, 2 of 253). The HCV strains involved in mixed gt1a/gt3 infections were dispersed throughout the phylogenetic trees (Figure 1) and were distinct from potential contaminants such as reference strains, other cohort strains and replicon strains cultured in nearby labs, thus confirming the presence of both genotypes. The average pairwise distances of the mixed genotype strains (gt1a, 0.176; gt3, 0.185) and strains from single genotype infections (gt1a, 0.182; gt3, 0.174) were comparable. By analysing sequencing chromatograms, it was suspected that one sample (gt1a/gt3 sample G30) contained multiple gt1a strains. Clonal analysis revealed two distinct gt1a strains (G30‐2 to G30‐6 in Figure 1) in addition to the coinfecting gt3 strain. Mixed genotype samples G3‐80 and G3‐125 were from the same individual collected 1 month apart with the more recent sample (G3‐125) obtained during treatment with an unspecified antiviral regimen predating DAA introduction. Although the gt3 strains at the two time points were identical, the gt1a sequences differed substantially (Figure 1).

### 3.3 Quantification by rt‐PCR

Genotype‐specific rt‐PCR assays were developed to quantify the relative proportions of gt1a and gt3 present in samples with mixed genotypes. Consistent detection of less than 10 copies/μL of RNA was observed for both gt1a and gt3 transcript controls whilst sample‐free controls were negative.

All 20 mixed genotype samples were assayed by genotype‐specific rt‐PCR to determine individual viral loads, and positive rt‐PCR results for both genotypes were obtained for 15 samples. In each sample, there was a major and a minor genotype, the latter constituting less than 21% of the combined viral load (Table 2) and in 10 of 15 samples constituting less than 1% of the combined viral load. Most (14 of 15) of the major genotypes have correlated with the clinical diagnosis of the patient. The exception was sample G3‐85 that was clinically diagnosed as a gt3 infection; however, the major genotype by rt‐PCR was gt1a which constituted 99.6% of the combined viral load. In the remaining gt1a‐diagnosed individuals with mixed infections (n = 2), the minor gt3 strains comprised 0.56% and 6.91% of the total viral load. In gt3‐verified samples, the minor gt1a strains comprised 0.01%‐21% of the combined viral load. The limit of detection of the PCR assays for samples with mixed genotypes was less than 58 IU/mL.

**Table 2:** Viral loads of gt1a and gt3 in mixed genotype infections

Sample	Gt 3 VL (copies/μL)	Gt 1a VL (copies/μL)	% gt3a	% gt1a	Major genotype^a^	Clinical genotype^b^
G‐27	5878	9.44	99.84	0.16	3	3
G45	393	10.65	97.36	2.64	3	3
G3‐36	27064.79	13.2	99.95	0.05	3	3
G3‐41	15479.58	19.75	99.87	0.13	3	3
G3‐67	34198.66	71.75	99.79	0.21	3	3
G3‐73	1020.66	265.27	79.37	20.63	3	3
G3‐80	30.24	3.55	89.49	10.51	3	3
G3‐85	9.92	2781.49	0.36	99.64	1a	3
G3‐125	627.31	44.37	93.39	6.61	3	3
G3‐128	20448.3	6.85	99.97	0.03	3	3
G3‐155	12557.81	2.75	99.98	0.02	3	3
G3‐166	44811.86	13.25	99.97	0.03	3	3
G3‐176	763.48	1.67	99.78	0.22	3	3
G1a‐92	55.69	13363.43	0.42	99.58	1a	1a
G1a‐128	71.79	967.27	6.91	93.09	1a	1a

VL, viral load.

a Major genotype as determined in this study.

b Infecting genotype determined clinically.

### 3.4 Analysis of mixed genotype infections by PCR‐NGS

The pan‐genotypic primers for the E1/E2 region (Table 1) were validated against an in‐house panel of 64 previously typed HCV samples spanning nine subgenotypes from five genotypes (Table S1). These primers were subsequently used for the PCR‐NGS amplification. Between 171 000 and 1 310 000 p.e., reads were produced from these samples of which 23 000‐825 765 mapped to the amplified HCV E1/E2 region (Table 3). The exceptions were sample G3‐71 (394 p.e. reads, 83 E1/E2 reads) and the spike‐in control Gt3‐H (6 042 524 p.e. reads, 2 276 655 E1/E2 reads). Suspected contamination was detected in four samples, a gt2 strain closely related to HC‐J6.D00944^24^ and gt1a sequences related to H77 (>98% homology), both used locally in cell culture studies. No other genotypes or subgenotypes were detected by PCR‐NGS.

**Table 3:** PCR‐NGS Gt1a and Gt3 reads

Sample name	Total no. of reads^a^	Total no. of E1‐E2 reads	Normalised Gt1a reads (%)^b^	Normalised Gt3 reads (%)^b^
Mixed infections				
G3‐36	763 960	168 114	0.0268	99.9732
G3‐41	516 764	198 175	0.5707	99.4293
G3‐44	539 726	229 862	0.0009	99.9991
G3‐51	560 912	185 930	3.5879	96.4121
G3‐67	515 456	140 949	0.0021	99.9979
G3‐73	804 876	391 168	0.0013	99.9987
G3‐80	681 740	47 351	0.0169	99.9831
G3‐128	559 940	161 272	0.4266	99.5734
G3‐155	526 418	160 504	0.0019	99.9981
G3‐166	528 010	154 295	0.2664	99.7336
G3‐176	597 822	347 419	17.5045	82.4955
E‐15	248 034	23 641	99.6447	0.3553
E‐49	587 092	123 546	0.6054	99.3946
G‐27	628 534	110 341	0.3471	99.6529
G‐30	642 162	184 349	0.0016	99.9984
G‐45	631 746	121 395	0.0025	99.9975
G1a‐128	413 560	371 392	90.1118	9.8882
G3‐85	633 624	346 956	99.9983	0.0017
G1a‐92	994 070	318 881	96.1403	3.8597
Gt1a mono‐infections				
E1a‐1	435 000	368 351	99.9240	0.0760
E1a‐2	394 130	296 101	99.9983	0.0017
E1a‐3	239 416	215 405	99.9986	0.0014
E1a‐4	282 276	257 764	99.9977	0.0023
E1a‐5	476 342	419 911	100.0000	0.0000
E1a‐6	351 616	314 002	100.0000	0.0000
E1a‐7	442 038	384 902	99.9964	0.0036
E1a‐8	491 074	393 892	100.0000	0.0000
E1a‐9	683 040	608 478	100.0000	0.0000
E1a‐10	512 770	432 815	100.0000	0.0000
E1a‐11	475 102	426 133	99.9991	0.0009
E1a‐12	264 866	236 048	99.9992	0.0008
E1a‐13	388 436	338 405	99.9994	0.0006
E1a‐14	320 212	291 889	99.9425	0.0575
E1a‐15	513 908	429 738	99.9998	0.0002
E1a‐16	670 778	591 941	99.9976	0.0024
E1a‐17	895 712	825 765	100.0000	0.0000
E1a‐18	1221 904	686 278	99.9985	0.0015
E1a‐20	366 268	338 870	99.9988	0.0012
Gt3 mono‐infections				
G3‐18	512 136	232 372	0.2780	99.7220
G3‐29	432 544	167 388	0.0125	99.9875
G3‐35	924 612	254 339	0.0712	99.9288
G3‐42	286 708	74 476	0.0081	99.9919
G3‐52	300 126	148 141	0.0007	99.9993
G3‐60	412 372	148 750	1.2208	98.7792
G3‐71	394	83	0	100
G3‐82	760 394	358 909	0.0014	99.9986
G3‐95	330 494	272 688	0.0238	99.9762
G3‐109	225 504	146 349	1.4930	98.5070
G3‐116	249 434	171 528	0.0006	99.9994
G3‐127	303 750	80 229	0	100
G3‐136	829 362	164 982	0.1109	99.8891
G3‐146	264 382	226 372	0.0009	99.9991
G3‐153	289 978	249 355	0.0056	99.9944
G3‐164	226 114	180 287	0.0011	99.9989
G3‐188	170 946	157 875	0.0215	99.9785
G3‐191	270 718	232 103	0.0009	99.9991
G3‐209	282 206	253 599	0.0016	99.9984
G3‐218	234 550	218 379	0	100
Controls				
Gt1a‐T1	1277 280	312 285	100	0
Gt1a‐T2	1302 070	302 439	100	0
Gt1a‐T3	1276 906	231 925	99.9987	0.0013
Gt1a‐H^c^	391 078	52 645	99.9506	0.0494
Gt1a‐M^c^	360 610	60 130	99.9684	0.0316
Gt1a‐L^c^	279 626	7328	99.8499	0.1501
Gt3‐T1	745 546	191 069	0.0063	99.9937
Gt3‐T2	553 894	121 431	0.0066	99.9934
Gt3‐T3	713 904	103 927	0.0048	99.9952
Gt3‐H^c^	6042 524	2276 655	47.5672	52.4328
Gt3‐M^c^	402 494	122 609	5.4686	94.5314
Gt3‐L^c^	174 596	43 101	0.5824	99.4176
Serum neg.1^d^	667 952	72	0.0006	0.0102
Serum neg.2^d^	472 938	101	0.0214	0

a The total number of paired‐end reads per sample.

b The number of reads for each genotype was normalised to the total number of E1‐E2 reads.

c Gt1a or Gt3a clonal transcripts with spiked‐in controls of the opposite genotype constituting 10% (H), 1% (M) or 0.1% (L) of the total amount (10^6^ copies/μL).

d Sera derived from individuals tested negative for HCV. Normalisation was performed against the total number of reads.

The reads were normalised against the total number of E1/E2 reads and expressed as a percentage. The percentages of normalised reads of the opposite genotype derived from the single genotype fidelity gt1a (gt3 < 0.002%) and gt3 (gt1a <0.007%) controls were low (Table 3). The serum negative controls, which were normalised against the total number of reads, also contained low levels of HCV E1/E2 reads (<0.03%). Based on the false‐positive figures from transcript and serum controls, a false‐positive threshold of 0.019% of reads was calculated (average percentage of normalised reads + 2 × standard deviation). For samples identified as gt1a mono‐infections by standard PCR, 2 of 19 (10.5%) contained gt3 reads above the false‐positive rate, whereas 7 of 20 (35%) of the gt3 mono‐infection samples contained gt1a reads. Gt3 major/gt1a minor infections were more abundant than gt1a major/gt3 minor infections, showing a trend (*P* = .063) similar to the PCR analysis. Mock mixed genotype controls comprised reads of the major and minor genotypes as expected; however, the actual percentages did not reflect the level of spike‐in (0.1, 1.0 and 10%) of the minor genotypes and were lower than expected for gt1a (0.15, 0.03 and 0.04%, respectively) but higher than expected for gt3 (0.58, 5.47 and 47.57%, respectively) indicating that the PCR‐NGS method was not quantitative.

Consensus sequences from the reads generated for the gt1a fidelity controls showed 100%, 98.8% and 98.7% fidelity, with all errors detected in genome positions 1552‐1563. The gt3 transcripts were 100% homologous to the original control sequence except in the region 1491‐1570 where there were several errors and a missing 30 bp region, likely the result of exclusion due to diminishing read quality at the ends of the reads.

The major genotypes identified by rt‐PCR within the mixed infection samples were all identified as such by NGS, including sample G3‐85 that was originally diagnosed as gt3 but was identified in this study by rt‐PCR and PCR as a gt1a major/gt3 minor sample. For the minor strains, the percentage of samples with normalised reads below the false‐positive threshold was 36.9% (7 of 19). It is therefore unlikely that these samples would have been identified as mixed infections by PCR‐NGS alone. There was no correlation (*R* ^2^ = 0.0013, data not shown) between the percentage of normalised reads of the minor strains and their percentage within the total viral load ascertained by rt‐PCR. The overall percentage of individuals with mixed infection in this subset of samples was similar for the NGS and PCR methods (36.2% and 34.5%, respectively), and the percentage of agreement between the methods was 72.4%. The number of samples testing positive for mixed infection by either NGS or PCR was 48.3%. If the gold standard test is considered to be detection of mixed infection by either PCR or NGS, then PCR (67.9%) and NGS (60.7%) had similar sensitivities. However, the subset used for the NGS consists of all the mixed infection samples detected by PCR^19^ and only a small proportion of the samples testing negative for mixed infection by PCR (39 of 487). Proportionally, the expected percentage of mixed infections by NGS from 506 samples is 24.4% ([(11 + [(9/39) × (506‐19)])/506] × 100). In this instance, sensitivity of the NGS method in terms of the gold standard is 91.8%, whereas the PCR assay is only 14.5%.

**Figure 2:**
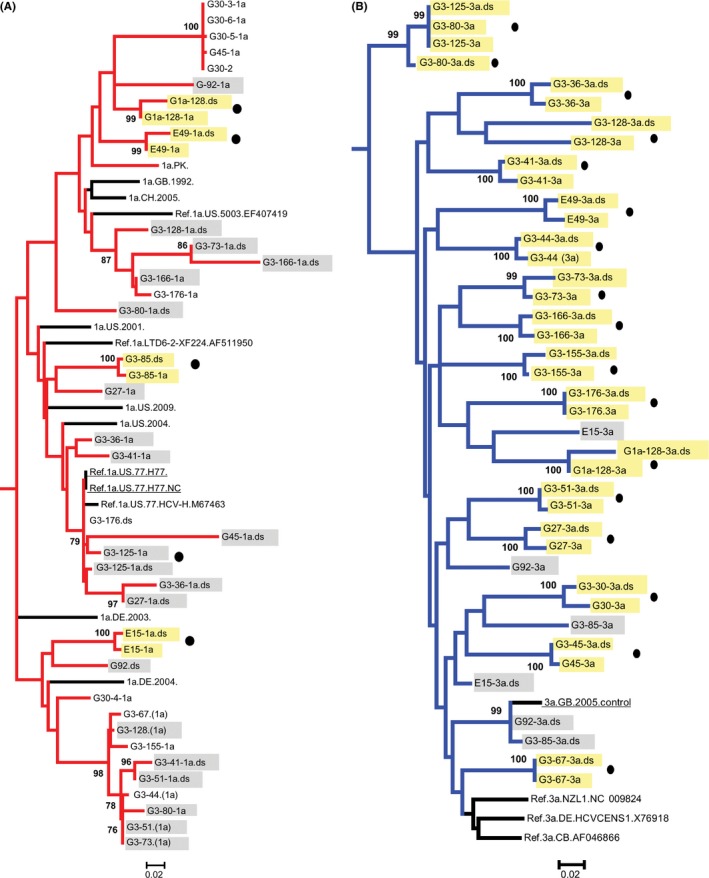
Maximum likelihood phylogenetic tree comparing A, gt1a sequences (red) and B, gt3 sequences (blue) sequences of mixed infection samples obtained by Sanger sequencing and consensus sequences from deep sequencing. Labels with the suffix.ds are the NGS consensus sequences. Control strains are underlined, and samples without a corresponding consensus sequence from deep sequencing are shown in red. Sequences from the same sample that cluster are highlighted in yellow, and those sequence pairs not clustering are highlighted in grey. Circles denote major strains (as determined by rt‐PCR). Bootstrap support of >70% after 1000 replicates is shown

A consensus of the NGS reads for each sample was compared phylogenetically to the Sanger sequences (Figure 2). Of the major genotypes, 18 of 20 NGS consensus sequences were identical or highly similar to the Sanger sequences. Considerable heterogeneity was observed between sequences obtained by the two methods for the other two samples (G1a‐92 and E‐15). There was little correlation between the minor genotype sequences produced by Sanger and NGS methods, with just 2 of 15 minor strain sequences (G1a‐128 and E‐49) clustering.

## 4 DISCUSSION

In this study, a genotype‐specific nested PCR targeting the E1‐E2 region was developed and used to screen HCV‐positive samples for the presence of mixed gt1a/gt3 infections. The mixed genotype infection prevalence rate in a cohort of 506 HCV‐positive individuals from Scotland previously diagnosed with either gt1a or gt3 infection was 3.8%. The nested PCR assay proved to be both sensitive and highly specific at the subgenotype level, capable of detecting low‐level secondary infecting genotypes in a high background of the major genotype. The E1‐E2 region is infrequently used for mixed HCV genotype screening except as part of a fragment >1000 bp in length,^25^ with the 5′ UTR and core regions being favoured. Whilst these regions can be used effectively for genotyping, diversity restriction means they are not always suitable for subtyping viral strains.^26^ The relatively short E1‐E2 region targeted in this study was highly discriminatory for genotyping and subgenotyping, providing more information than current clinical testing protocols.

The rate of mixed HCV genotype infections identified by PCR in our cohort (3.8%) is similar to the low prevalence rates observed in studies with large cohort sizes.^5^, ^27^ Studies involving smaller cohorts^1^, ^28^, ^29^ tend to have greater rates of HCV coinfection prevalence. The stringent focus on gt1a and gt3 may have contributed to the low prevalence rates observed. Gt1a and gt3 are the most common genotypes within the UK and are estimated to be responsible for 90% of all HCV infections.^30^ As mixed genotype infections involving a wide range of different genotypes have been documented,^31^, ^32^ the true prevalence of mixed genotype infection may be higher if all genotypes were analysed. However, no other genotypes were detected in our samples by PCR‐NGS, therefore it is unlikely that any such increase in prevalence would be substantial. A large proportion of the individuals within our cohort had a known history of injecting drug use which entails a significantly higher exposure to multiple HCV challenges than other routes of HCV infection.^1^, ^29^, ^32^ Recent studies have also indicated that there may be a higher prevalence of superinfection and reinfection occurring within populations of PWID than is currently estimated and that factors such as long testing intervals and rapid viral clearance are hindering detection.^33^, ^34^, ^35^ Most patients provided a single sample giving a snapshot of the course of the infection. As HCV viral loads in chronically infected individuals can fluctuate substantially over the course of infection,^1^, ^31^ the relative proportions of genotypes in a coinfection may not be static and multiple genotype infections can be transitory in nature.^36^, ^37^ Diagnostically, it is difficult to differentiate between acute and chronic infection and it is therefore unclear if either genotype within the mixed infections of this cohort is transitory or if both genotypes had established chronic infections. The latter situation is more likely as most of our cohort were unlikely to be actively injecting drugs at the time of sampling; the average age of subjects within our cohort was 42.6 ± 9.8 years, the average age at which injecting habits develop are 21‐22 years of age 38, 39 and the average injecting career length has been estimated to be 8 years.^40^

The genotype‐specific rt‐PCR assay was less efficacious than the nested PCR for the mixed infection samples, quantifying only 75% of the minor genotypes. The use of a non‐nested protocol may explain the reduced sensitivity of the rt‐PCR protocol compared to the screening assay; however, the assays displayed similar sensitivities with control samples. Alternatively, the rt‐PCR specificity at the subgenotype level may account for the discrepancies. It is notable, however, that most of the samples where the minor genotype was not detected by rt‐PCR were of older origin and had undergone several freeze‐thaw cycles following PCR screening, potentially affecting RNA yield.

Results from the genotype‐specific rt‐PCR assay indicated the HCV population structure comprised a major and a minor genotype. A significantly greater rate of mixed infection was determined in individuals diagnosed with gt3 than in patients diagnosed with gt1a. The disproportionate rate of individuals clinically diagnosed with gt3 infections with mixed infections could suggest a difference in sensitivity between the two genotypic rt‐PCR assays; however, this was not apparent when quantifying the transcript controls in mock mixed genotype infections. Alternatively, the primers used to amplify gt3 may not have sufficient broad coverage of the genotype. This seems unlikely as all of the mono‐infected gt3 strains were amplified, and there is no evidence that a large proportion of gt3 infections are currently undiagnosed, which would occur if this were the case. We cannot discount, however, that gt3 strains involved in mixed infections are phylogenetically divergent from mono‐infection strains and have poor primer coverage. Gt1 infections can be more difficult to treat with non‐DAA treatments than other HCV genotypes,^41^, ^42^ and as 28.2% of individuals in our study had been previously treated without achieving an SVR, there may have been partial resolution of coinfecting genotypes^11^, ^43^ in some treated individuals which has disproportionally resolved gt3 minor strains. The rate of infection among drug users who are already anti‐HCV positive is lower than individuals previously unexposed, suggesting there may be some form of partial immunity,^44^, ^45^ and it is possible that some genotypes confer a broader cross‐protective immunity than others. Minority HCV strains within a superinfection may survive by replicating within extrahepatic sites,^46^ and there may be genotypical differences in ability to adapt for survival in these regions. The reasons for the disparity in coinfecting rates of the genotypes are likely highly complex and involve a combination of genotype‐specific host response^31^ and viral competition.

A pan‐genotypic primer set developed for the PCR‐NGS proved to be highly effective at amplifying and typing gt1, gt2, gt3 and gt4 strains and one gt6a isolate at the subgenotype level. Despite this, no genotypes other than gt1a and gt3 were detected with the exception of assumed contaminant gt2 strains which were highly similar to a replicon strain used locally. To ascertain the suitability of this assay for clinical diagnostics, it would be necessary to test the primers against gt5 and gt7 as well as more gt3 and gt6 subtypes. Data collected during assay optimisation suggested that the primers could function effectively with at least two known mismatches.

PCR‐based deep sequencing was selected in preference to metagenomic methods as the low ratio of HCV to human RNA affects the sensitivity of the latter method. HCV has been detected in clinical samples by metagenomic methods at levels as low as 2000 IU/mL^47^; however, this is still substantially less sensitive than would be required to detect most minor strains we identified by PCR. Sequencing errors and PCR bias can be problematic in PCR‐NGS. The sequencing errors we identified using fidelity controls were restricted to the centre of sequences, equating to the end of reads in paired‐end sequencing, where sequence quality often deteriorates. This issue could be easily resolved using an Illumina 500 cycle format. The PCR bias we observed, as demonstrated by poor agreement between minor strain proportions and read numbers in mock mixed infections, indicates that the assay cannot be used for quantification. Rates of frequency can be distorted by margins of up to 100‐fold relative to the true frequency.^48^, ^49^

For the sample subset that was deep sequenced, the percentage of mixed infections were similar between the methods and, in comparison with detection by either PCR or NGS which we designated as the gold standard, the individual methods had similar sensitivities. However, an estimate of the expected percentage in the original sample set suggested a mixed infection prevalence rate of 24.4% by PCR‐NGS, much greater than the rate calculated from PCR analysis (3.8%). A key issue in interpreting NGS data for viral diagnostics lies in defining background contamination. We applied a false‐positive threshold based on background reads obtained in negative controls. However, there is uncertainty as to the reliability of such methods for PCR‐NGS which has been shown to be poorly quantitative, in this study by inaccurate read proportions obtained from mock mixed infections, and elsewhere.^47^, ^48^, ^49^ Reduction in PCR cycles may improve the quantitative aspect of PCR‐NGS.

In conclusion, the prevalence rate of mixed infection in this UK cohort of 506 individuals by PCR was 3.8%, with gt3 as the major genotype in most samples. The mixed infection rate obtained from PCR‐NGS data was much higher; however, interpretation is hampered by the designation of false‐positive thresholds with a technique that is poorly quantitative.

## CONFLICT OF INTEREST

The authors have no competing interests.
