# Supplementary material for: Prevalence of mixed genotype hepatitis C virus infections in the UK as determined by genotype‐specific PCR and deep sequencing
Source: J Viral Hepat. 2018 Feb 21;25(5):524–34. doi: 10.1111/jvh.12849 (PMC5947153; doi:10.1111/jvh.12849)
Supplement: Supplementary file 1 [file JVH-25-524-s001.docx]

**SUPPLEMENTARY TABLE** Validation of pan-genotypic primers

| **Reported Genotype** | **Typing Results** | **Number Tested** |
| --- | --- | --- |
| 1a | 1a | 20 |
| 1b | 1b | 8 |
| 1c | 1g^a^ | 1 |
| 2 | 2a | 5 |
|  | 2b | 4 |
| 3 | 3a | 20 |
| 4 | 4a | 3 |
|  | 4d | 2 |
| 6 | 6a | 1 |

^a^ There was disagreement between the clinically reported genotype and the genotyping performed in this study for one sample. Phylogenetic analysis suggested the sample is Gt1g.
